# Nutritional status, immunonutrition, and gut microbiome: a coming of age for immunotherapy?

**DOI:** 10.3389/fimmu.2025.1612567

**Published:** 2025-08-25

**Authors:** Elisa Mattavelli, Francesco Agustoni, Alice Tartara, Francesca De Simeis, Lorenzo Perrone, Riccardo Caccialanza, Paolo Pedrazzoli, Valentina Da Prat

**Affiliations:** ^1^ Clinical Nutrition and Dietetics Unit, Fondazione IRCCS Policlinico San Matteo, Pavia, Italy; ^2^ Department of Internal Medicine and Medical Therapeutics, University of Pavia, Pavia, Italy; ^3^ Medical Oncology Unit, Oncology Department, Fondazione IRCCS Policlinico San Matteo, Pavia, Italy

**Keywords:** nutritional status, body composition, immunonutrition, gut microbiome, immunotherapy, treatment response and efficacy

## Abstract

In the last decades, immunotherapy has revolutionized cancer treatment. Despite its success, a significant number of patients fail to respond, and the underlying causes of ineffectiveness remain poorly understood. Factors such as nutritional status and body composition are emerging as key predictors of immunotherapy outcomes. In particular, poor nutritional status, sarcopenia, and low skeletal muscle mass are associated with poorer survival and immunotherapy response in several cancers. Conversely, certain parameters of body composition, such as adiposity, may have beneficial effects on immunotherapy efficacy. Nutritional status and body composition can be targeted through tailored nutritional support, making it a potential strategy to improve immunotherapy outcomes. Specific nutrients and modulation of the gut microbiota may further enhance immune functions, offering promising avenues for clinical improvement. Despite the promising potential of tailored nutritional support, clinical evidence remains limited, and further research is needed to establish optimal strategies to optimize immunotherapy response and effectiveness.

## 1 Introduction

The history of immunotherapy dates back to the early 1980s, when William Coley harnessed the immune system to treat bone cancer (1). This is a treatment modality based on the mobilization of the immune system in order to recognize and destroy tumor cells. Immune checkpoint inhibitors (ICIs) have been developed with the intent to act on those pathways of “self-tolerance” used by tumors to escape recognition and then destruction by the immune system (2). Key negative regulatory pathways or ‘checkpoints’, such as the PD-1/PD-L1 pathway, physiologically control auto-reactivity, and the improved understanding of their involvement in cancer is revolutionizing tumor immunotherapy. The novel agents that target immune checkpoints, by releasing the ‘brakes’ on pre-existing tumor-reactive T cells and facilitating the generation of new T cell responses, have led to impressive clinical benefits across a number of different tumor types. ICIs, targeting cytotoxic T-lymphocyte antigen 4 and programmed cell death protein 1 or its ligand (anti-CTLA-4, anti-PD-1, and anti-PD-L1 drugs, respectively) are the best-known type of immunotherapy (2). However, other promising approaches are emerging against other potential immune check-points such as T cell immunoreceptor with immunoglobulin and ITIM domain (TIGIT), immune checkpoint receptor lymphocyte-activation gene 3 (LAG-3), CD73, NKG2A, or adoptive T-cell therapy and chimeric antigen receptor (CAR) T-cell (3–5).

However, many patients still do not experience clinical benefits from these therapies, and indeed some tumor types appear particularly resistant (6). On the other hand, an exciting component of novel immunotherapeutic strategies is the durability of clinical benefit observed, with some patients achieving disease control for many years.

To date, several factors have been identified as influencing immunotherapy efficacy, such as PD-L1 expression, tumor mutational burden, effective T-cell infiltration, TGF-β activity, previous treatments and tumor proliferative potential (7).

Characterization of immunological competency, evaluation of direct antitumor immune response and identification of baseline immunological parameters correlated with clinical benefit, could be instrumental in the selection of potential responders to ICIs and, through a further insight into their mode of action, in the design of clinical trials and assessment of treatment response.

**Figure 1:**
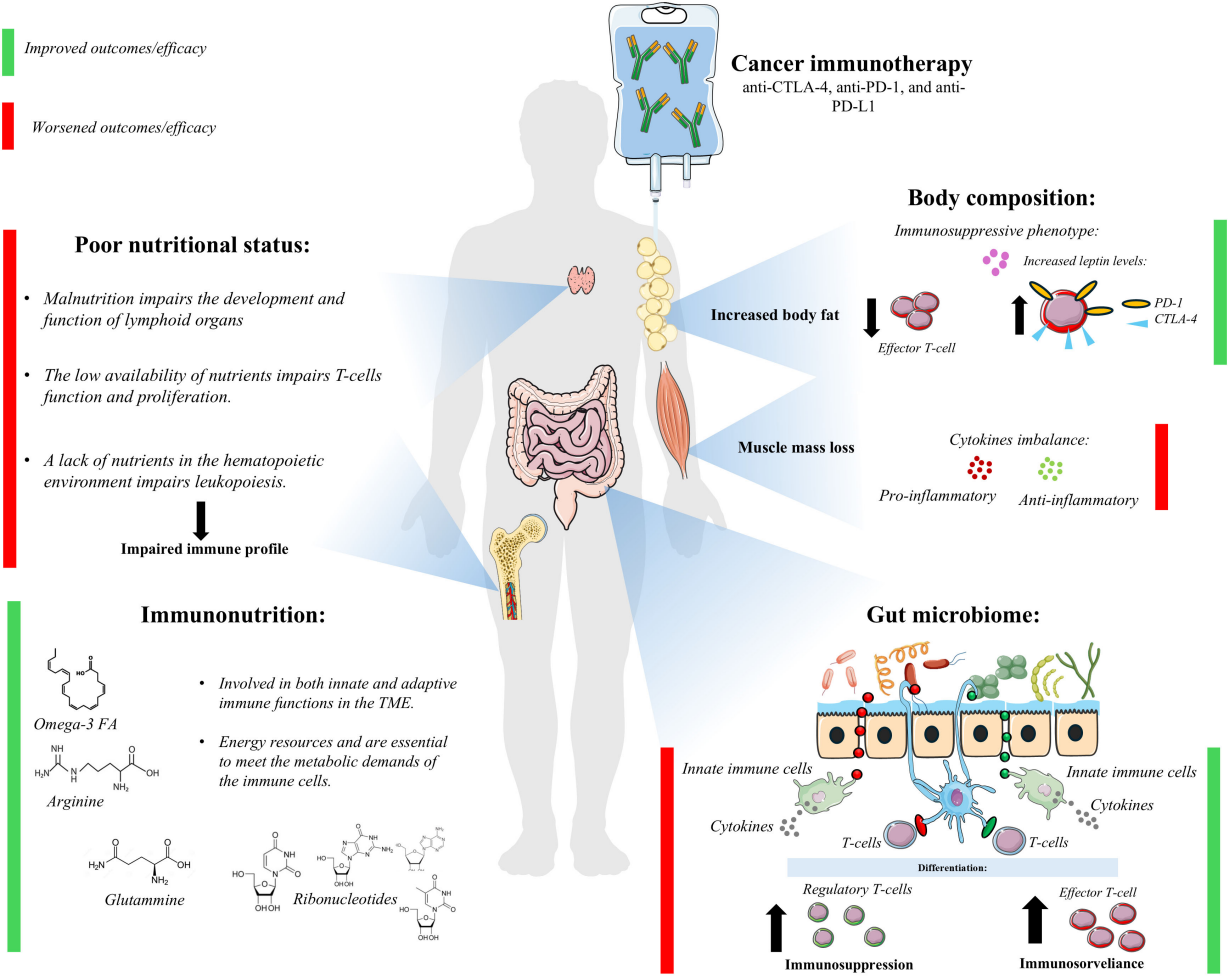
Nutritional aspects related to immunotherapy outcomes and efficacy.

Interestingly, poor nutritional status and impaired body composition are emerging as potential prognostic biomarkers to predict patient response to immunotherapy and thus improve treatment outcomes (8). In addition, nutritional support could be tailored to include specific nutrients that may improve the effectiveness of immunotherapy. Immunonutrition, which involves the use of specific nutrients (omega-3, arginine, and RNA nucleotides) to modulate the immune response, could play a key role in optimizing immunotherapy efficacy and patient outcomes. Modulation of the gut microbiota, which is known to influence immune function, might further enhance the effectiveness of immunotherapy by promoting a more favorable immune environment (**Figure 1**).

## 2 The prognostic role of nutritional status in patients receiving immunotherapy

A growing body of evidence supports the critical role of adequate nutrition in immune system function (9, 10).

Recent findings suggest the prognostic value of nutritional status in patients undergoing immunotherapy, regardless of cancer type. A low Prognostic Nutritional Index (PNI), defined as serum albumin (g/dL) multiplied by ten plus total lymphocyte count multiplied by 0.005 (11) and reflecting poor nutritional status, has been shown to predict reduced progression-free survival (PFS) and overall survival (OS) in advanced head and neck squamous cell carcinoma (HNSCC) patients receiving immunotherapy (12). Although younger age predicted better OS, the association between poor nutritional status and worse outcomes was independent of this factor (12). Similarly, in non-small cell lung cancer (NSCLC) patients treated with immunotherapy, lower baseline PNI, reduced body mass index (BMI), and malnutrition (as defined by French authority Guidelines, considering the percentage of weight loss in a fixed period and BMI values) were associated with worse OS, PFS and overall response rate (ORR) (13–16). In these cases, the associations were independent of other factors related to nutritional status and worse outcomes, including tumor stage (14), age, and performance status (15, 16) Furthermore, the independent association between poor nutritional status and worse immunotherapy outcomes holds true for patients diagnosed with advanced biliary tract cancer (17), advanced gastric cancer (18) and hepatocellular carcinoma (HCC) (19). Indeed, a recent meta-analysis including 663 patients with different cancer types who were treated with immunotherapy confirmed the prognostic value of poor nutritional status assessed by the Controlling Nutritional Status (CONUT) score, defined as the summary of points singularly attributed to albumin, lymphocytes counts and cholesterol levels according to pre-defined cut-off values (20) with worse OS, PFS, ORR and disease control rate (DCR) (21). A large retrospective study by Tang W. et al. highlighted that immunotherapy-treated patients who were at nutritional risk (Nutritional Risk Screening-2002 ≥ 3) had worse PFS, OS, and ORR; this association was independent of patients’ performance status (22).

Although the association between poor nutritional status and worse therapeutic outcomes appears to be independent of potential confounding factors, a more in-depth consideration of the immuno-inflammatory component is warranted. Most studies have assessed nutritional status using the two most applied indices: the PNI and the CONUT score. However, both indices include lymphocyte count in their scoring systems, thereby incorporating not only strictly nutritional aspects but also immuno-inflammatory components. This overlap may limit our ability to isolate the specific impact of nutritional status on immunotherapy outcomes. Nonetheless, some studies have demonstrated that nutritional deterioration alone is associated with poorer outcomes. For instance, Tang W. et al. reported that an NRS-2002 score ≥3 was independently associated with worse outcomes, regardless of systemic inflammatory index, lymphocytes, and C-reactive protein (22). A similar finding has been observed with the CONUT score, whose values were found to be independent of inflammatory indexes (18). Interestingly, the combination of low BMI and an elevated platelet-to-lymphocyte ratio has been associated with significantly worse overall survival outcomes (13). Together, these findings suggest that jointly evaluating nutritional and inflammatory status in patients undergoing immunotherapy may offer more comprehensive prognostic insights.

In addition to baseline assessment, dynamic monitoring of nutritional status throughout the treatment course is essential. In fact, treatment-related side effects, such as nausea, anorexia, and vomiting, along with the catabolic effects of cancer itself, can lead to reduced oral intake, further compromising nutritional status and exacerbating immune dysfunction, leading to impaired treatment efficacy and reduced overall prognosis (23). Guller M. and colleagues reported that in patients with stage IV NSCLC, a decrease of ≥2% in BMI was associated with worse survival and reduced response to immunotherapy (12). Intriguingly, baseline BMI was not found to be a prognostic factor for worse outcomes in this study (12). This scenario was also reported by Fang Q. et al., where only the decrease in nutritional indices, indicating a deterioration in the nutritional status of patients, but not their baseline values, was related to reduced overall survival (24). Similarly, the dynamic variations in BMI outperformed the prediction of ICIs response and efficacy compared to baseline BMI in 629 patients with advanced-stage cancers (25).

## 3 The prognostic role of body composition in patients receiving immunotherapy

Nutritional status is a multifaceted concept that extends beyond traditional markers such as BMI, albumin levels, or PNI. Among its key components, body composition has emerged as a critical determinant of clinical outcomes in immunotherapy.

Low skeletal muscle index (SMI) and myosteatosis were reported to be predictive of worse overall survival in HCC patients treated with immunotherapy. In the same cohort, increased subcutaneous and visceral adipose tissue was associated with longer survival (26, 27). Similar findings were reported in advanced NSCLC patients, where baseline presence of sarcopenia and myosteatosis, but not sarcopenic obesity, were independent predictors of OS, PFS, and ORR (28, 29). NSCLC patients with sarcopenia also experience reduced DCR, as reported by Li S. et al. (30). Low skeletal muscle mass was predictive of reduced OS but not of reduced PFS and increased occurrence of immune-related adverse events (iAEs) in melanoma cancer patients receiving immunotherapy (31, 32).

Also, not only reduced lean and muscle mass but also reduced subcutaneous adipose tissue was found to be predictive of worse OS in NSCLC and melanoma patients (33). The prognostic role of body composition was also confirmed in patients with other types of cancers undergoing ICIs and CAR T-cell treatment, where sarcopenia and low muscle mass were associated with worse PFS and OS (34).

## 4 The biological basis for the influence of nutritional factors on the immune response

The link between nutrition and the immune system has long been recognized (35). Interestingly, impaired nutritional status is considered the most common cause of immunodeficiency worldwide (36). The exact nature of immunodeficiency in undernutrition/malnutrition remains uncertain, but deficiencies of macro- and/or micro-nutrients are known to adversely impact both innate and adaptive immunity (10, 37, 38).

In particular, the lack of nutrients in the hematopoietic microenvironment impairs several biological processes, including leukopoiesis (39, 40). Malnutrition also impairs mucosal immunity and the humoral immune response (immunoglobulin A secretion) (38, 40–42). Moreover, malnutrition in early childhood alters the development and function of lymphoid organ (e.g. thymus) resulting in an imbalance of circulating mature and immature T- cells. Similarly, T cells are highly sensitive to nutrient availability; thus, nutritional status can have a significant impact on T cell proliferation, cytokine production, and immune responsiveness (43).

Muscle wasting is frequently associated with inflammatory cytokine imbalance, which could affect the efficacy of immunotherapy (44). Evaluating the prognostic role of body composition in cancer treatment has also brought attention to the obesity paradox. The finding that increased visceral and subcutaneous adipose tissue is associated with improved survival was also confirmed in cancer patients undergoing immunotherapy (27, 45–48). The metabolic abnormalities related to increased adipose tissue, such as increased blood cholesterol and leptin resistance, as well as genetic polymorphisms that result in the obese phenotype, affecting both metabolic and inflammatory pathways, are potential factors that influence the efficacy of immunotherapy. Obesity is frequently associated with chronic systemic inflammation and altered immune profile (49). Alden S. et al. reported a reduced number of T effector cells and an increased expression of PD-1 and CTLA-4 in obese compared to non-obese cancer patients (50). This immunosuppressive phenotype may contribute to tumor development and progression by facilitating immune evasion. Conversely, it may also represent a key factor for improved responsiveness to immunotherapy (51). Leptin, which is commonly elevated in obesity, is capable of upregulating PD-1 and PDL-1 expression, thus providing a broader target for ICIs (52). From a biomolecular perspective, this effect appears to be mediated by the activation of the nuclear transcription factor STAT3 following the interaction between leptin and its receptor. In CD8^+^ T cells, nuclear STAT3 can bind to the promoter region of the PD-1 gene, promoting its transcription, subsequent translation into protein, and expression on the cell surface (53). However, Frack M. et al. were not able to confirm the presence of a higher response rate to immunotherapy in obese patients (54). Of note, adipose tissue is divided into two main compartments: visceral adipose tissue (VAT) and subcutaneous adipose tissue (SAT). These two compartments exhibit distinct metabolic and inflammatory activities. VAT harbors a higher inflammatory potential with elevated secretion of MCP-1 and PAI-1, and pro-inflammatory ecoisanoids and cytokines (IL-6) compared to SAT (55). Moreover, despite the limited sample size, VAT and SAT display distinct leptin secretion profiles, indicating that SAT may be the primary source of its production (56, 57). Therefore, the response to immunotherapy might depend on the predominant adipose tissue compartment. To date, most epidemiological studies report improved immunotherapy efficacy in the context of both high SAT and high VAT. Nevertheless, these findings may not fully reflect the underlying biological reality. While certain factors, such as gender, influence the accumulation of SAT versus VAT, it is difficult to distinguish the specific effects of each due to their high correlation. One approach to better understanding this association could be implementing further stratification based on individual VAT and SAT levels in clinical trials. In this context, the use of flat dosing regimens raises additional concerns. This approach does not account for body composition, which is particularly problematic for patients with high adiposity and reduced muscle mass. Given their potential role in influencing drug distribution and its metabolism, a uniform dosing approach may not be optimal to maximize treatment efficacy.

## 5 Nutritional approaches to improve immunotherapy outcomes: where do we stand?

Nutritional approaches directed to modifiable patient-related factors, such as nutritional status and body composition in patients treated with immunotherapy, could improve the treatment efficacy, which makes them particularly interesting compared to other disease-related factors (e.g., PD-L1 expression).

In general, nutritional support is a cornerstone of treatment in cancer patient care. Current guidelines emphasize the importance of early nutritional support to prevent deterioration and promote improvement of patient nutritional status, which is associated with treatment response and survival (58). However, in the context of immunotherapy, nutritional management remains an often overlooked aspect, as evidenced by the paucity of clinical trials evaluating the effects of nutritional support in this setting (59). Nutritional support could have a dual benefit: on the one hand, it may ensure the maintenance or improvement of nutritional status, allowing the patient to meet the energy and protein needs; on the other hand, as some nutrients interact with the immune system, it could potentially enhance the effectiveness of immunotherapy (60). Integrating these two elements could maximize the therapeutic benefit and improve the quality of life for cancer patients. Recent research has made significant progress in identifying the biological mechanisms by which certain nutrients interact with key players in the immune system, such as T cells involved in the elimination of tumor cells (61). For example, vitamin D is involved in the regulation of immune function and vitamin D receptor is found in immune cells (62). Zinc is also essential as it regulates several metabolic pathways in both adaptive and innate immune cells (63). Selenium, an essential component of selenoproteins, is also involved in immune processes (64, 65).

Immunonutrition refers to the intake of specific nutrients, such as omega-3 fatty acids, arginine, glutamine, and nucleotides that modulate the immune response (66). In particular, omega-3 fatty acids play a role as precursors of pro-resolving mediators, thus reducing the pro-inflammatory response. They are involved in innate immune functions, and affect T-cell functions (67–69). In addition, some preclinical evidence suggests a role for omega-3 fatty acids in suppressing tumor growth through gene regulation mechanisms in NK cells, promoting their activation, enhancing NK cell-induced cancer cell apoptosis, and protecting them from degradation (70). Omega-3 fatty acids also reduce tumor growth by activating Th1 cells and increasing eosinophil recruitment in the tumor microenvironment (71). Arginine and glutamine exert immunostimulant effects, promoting T-cells activities and reducing pro-inflammatory cytokines (interleukin-1, interleukin-6, and tumor necrosis factor-alfa) (67, 68, 72). The metabolic requirements of immune cells depend on the availability of these two amino acids; therefore, their depletion negatively affects both natural and therapy-induced anti-tumor immunity (72–74). Nucleotides are the essential components for DNA and RNA biosynthesis and are an energy resource for the immune system, thus playing a key role in immune cell proliferation (68). Moreover, immune cells have nucleotide-specific receptors on their cell membranes, and their activation plays a critical role in anti-cancer innate and adaptive immune responses (75).

## 6 Alternative nutritional approaches in cancer immunotherapy

Along with the development of immunonutrition formulations, some dietary patterns have gained interest in the search for nutritional approaches that might enhance immunotherapy efficacy. To date, numerous dietary patterns have been assessed, mainly in preclinical models, suggesting that macronutrient modification (e.g., ketogenic diets, low-protein diets, and high-fiber diets), but also caloric restriction, might improve the anti-tumor immune response by regulating the infiltration and function of T-cells, thus improving immunotherapy effectiveness (76).

Ketogenic diets could promote weight loss, potentially worsening patients’ nutritional status through several mechanisms, such as limited food choices, reduced palatability, and increased satiety (77). High-fiber diets to modulate gut microbiome composition and calorie-restricted diets could promote reductions in daily calorie intake, making patients more susceptible to developing impaired nutritional status if they do not meet their energy requirements (78). In addition to the detrimental effects that these diets may have on patients’ nutritional status, which is a prognostic factor in immunotherapy, future clinical trials should also consider the psychological impact of prescribing restrictive diets, an aspect that remains underexplored (79).

## 7 Gut microbiome and immunotherapy: challenges to be addressed for personalized approaches

The highly individualized response to immunotherapy suggests that even providing adequate nutritional support to maintain or improve a patient’s nutritional status may not result in the same improvement in immunotherapy response in all cancer patients (80). Among the individual factors that affect immunotherapy efficacy, emerging research highlights the role of the gut microbiome and its metabolites both as prognostic factors and potential therapeutic targets (81–83).

To date, several gut microbiome signatures and gut microbiome-derived metabolites have been identified to influence immunotherapy response and clinical outcomes (83–86). However, these features remain inconsistent across studies, making it difficult to establish a definitive set of gut microbiome biomarkers that reliably predict immunotherapy response in cancer patients. Several aspects may explain this, including: i) the redundancy and overlapping roles of bacterial species in modulating immune pathways, ii) methodological issues related to the type of analysis (e.g. 16S rRNA *vs*. whole metagenomics sequencing) which can significantly affect the sensitivity in identifying gut bacteria, and iii) the influence of environmental factors (e.g., geographic location, diet, physical activity level) on gut microbiome composition and metabolite production in different populations (80). Interventions aimed at modifying the microbiome and its metabolites may also provide valuable insights. Indeed, modulation of the gut microbiome through fecal microbiota transplantation may affect the efficacy of immunotherapy by activating the immune response (87), but some safety issue still need to be addressed. First of all, the complete transfer of another individual’s microbiome may inadvertently introduce predispositions to other diseases, as the microbiome is implicated in various pathological phenotypes beyond cancer (88). Probiotics may represent a safer alternative to modulate the gut microbiome to enhance the efficacy of immunotherapy, but the variability in patient response to probiotic administration and the potential adverse effects underscore the importance of continued research in this area (89).

## 8 Current clinical trials investigating nutritional approaches and the microbiome in cancer immunotherapy

Several studies are assessing the beneficial effects of immunonutrition in radio- and chemotherapy (NCT06349148, NCT06085365, NCT05892354, NCT04611113 (90)). However, to date, no studies have investigated the therapeutic efficacy of immune nutrient-enriched blends in patients with solid tumors undergoing immunotherapy treatments. Currently, only two clinical trials are assessing the effectiveness of combining immunonutrition and immunotherapy. The NCT05384873 trial aims to evaluate the efficacy of early systematic provision of oral nutritional supplements enriched with immunonutrients in patients with NSCLC undergoing immunotherapy and receiving nutritional counselling, with improving of progression-free survival (PFS) as primary end-point (91). The NCT06342167 trial, a single-arm, multi-center Phase II study, to evaluate the efficacy and safety of combining immunotherapy plus radiotherapy with immunonutrition in patients with locally advanced esophageal squamous cell carcinoma (**Supplementary Table 1**).

In contrast, a larger number of studies are investigating the role of gut microbiome composition in immunotherapy response (**Supplementary Table 2**). Some trials aim to identify microbiome signatures that may predict responses to immunotherapy (NCT06714903, NCT06050733, NCT06318507, NCT04638751, NCT04957511, NCT06613308, NCT04291755, NCT06709651 and NCT04107168). Others are exploring whether modulation of the gut microbiome through various strategies such as antibiotics (NCT05462496), probiotics (NCT05865730, NCT06428422), dietary interventions (e.g., Mediterranean diet: NCT06236360; high-fiber diet: NCT04866810, NCT06466434; prebiotic food: NCT06298734), or fecal microbiome transplantation (NCT06403111, NCT05251389, NCT05502913, NCT04163289, NCT05690048, NCT05750030, NCT03772899), can improve the effectiveness of immunotherapy.

Regarding the effects of alternative dietary approaches on immunotherapy outcomes, two ongoing interventional clinical trials are evaluating the effects of calorie-restricted diets on immunotherapy effectiveness in patients with NSCLC (NCT05703997) and breast cancer (NCT06033092) who are not at risk for malnutrition. A study is evaluating the ketogenic diet in patients with a BMI >18.5 and renal cell carcinoma (NCT06391099). Finally, a trial is assessing the effects of a low-protein diet in patients without malnutrition (ECOG performance status >2) with epithelial ovarian cancer (NCT05356182) (**Supplementary Table 3**).

Although numerous studies are addressing the microbiota as a prognostic factor or modifiable element to improve immunotherapy efficacy, there is a clear need for more research focused on nutritional approaches, including nutritional status, immunonutrition, and alternative dietary patterns, in the context of immunotherapy.

## 9 Conclusions

Although significant progress has been made regarding the role of nutritional status and immunonutrition in the context of immunotherapy, some aspects still need to be addressed.

Emerging evidence on the importance of nutritional status and body composition in patients undergoing immunotherapy highlights how these factors can significantly influence clinical outcomes. Nutritional parameters and body composition have been identified as prognostic factors, highlighting the fundamental role of early and prompt nutritional risk screening, accompanied by a detailed nutritional assessment when appropriate. This is essential in the context of immunotherapy, both to predict the prognosis of cancer patients and to identify those who would benefit the most from tailored nutritional support. However, the ability to improve treatment outcomes through tailored nutritional support is still under investigation.

Despite the growing interest in immunonutrition and the potential of alternative dietary approaches to enhance immunotherapy effectiveness, research on personalized nutritional strategies remains limited. Most of the randomized registrative clinical trials investigating immunotherapy do not include analysis of the nutritional status and body composition at baseline or nutrition-oriented endpoints. In particular, ongoing clinical trials investigating nutritional interventions and their role in optimizing immunotherapy outcomes with specific design and outcomes are essential to provide clearer evidence. Future studies should aim to better understand the relationship between nutritional status, nutritional support and immunotherapy response, focusing on personalized and targeted interventions to maximize treatment efficacy and improve patient quality of life. In this context, considering and modulating the composition of the gut microbiome and its metabolites may be a successful strategy in the future.

To drive meaningful progress, some mechanistic questions should be prioritized in preclinical models. These include investigating the effects of immunomodulatory nutrients on key immune cells and exploring the interaction between body composition and immunotherapy pharmacokinetics. Biomarker-driven pilot trials stratifying patients by immunophenotypes and body composition to evaluate targeted interventions like immunonutrition and microbiome modulation will be essential to improve clinical outcomes. Finally, health-economic analyses assessing the clinical and economic impact of early nutritional screening and targeted interventions in immunotherapy are needed to optimize patient care, and support the integration of dedicated nutritional programs, but also to achieve efficient healthcare resource management.

## Data Availability

The original contributions presented in the study are included in the article/**Supplementary Material**. Further inquiries can be directed to the corresponding author.
