# Supplementary material for: Nutritional status, immunonutrition, and gut microbiome: a coming of age for immunotherapy?
Source: Front Immunol. 2025 Aug 25;16:1612567. doi: 10.3389/fimmu.2025.1612567 (PMC12414764; doi:10.3389/fimmu.2025.1612567)
Supplement: Supplementary file 1 [file DataSheet1.docx]

# Tables

**Table 1: Ongoing clinical trial investigating the effectiveness of combining immunotherapy to immunonutrition.**

| **Title and ID number** | **Patients** | **Study design** | **Primary outcome/s** | **Secondary outcomes** |
| --- | --- | --- | --- | --- |
| Immunonutrition for Improving the Efficacy of Immunotherapy in Patients With Metastatic Non-small Cell Lung Cancer (MURAL)  NCT05384873 | Metastatic NSCLC | Randomized interventional study  Intervention: Immunonutrition  Control: standard ONS | - PFS | - Duration response - Treatment side effects - Body composition - Fatigue - QoL - Activity levels - Levels of immunological markers |
| Efficacy and Safety of Concurrent PD-1 Inhibitor and Radiotherapy With Immunonutrition for Esophageal Squamous Cell Carcinoma  NCT06342167 | Esophageal Squamous Cell Carcinoma | Single-arm interventional study  Intervention: IO+ RT+ immunonutrition support | - PFS | - ORR - OS - AEs incidence |

NSCLC: Non-Small Cell Lung Cancer; PFS: Progression Free Survival; QoL: Quality of Life; ORR: Objective Response Rate; OS: Overall Survival; AEs: Adverse Event; IO: immunotherapy; RT: Radiotherapy.

**Table 2: Ongoing clinical trial investigating the association between gut microbiome composition and immunotherapy.**

| **Title and ID number** | **Patients** | **Study design** | **Primary outcome/s** | **Secondary outcomes** |
| --- | --- | --- | --- | --- |
| Effect of Gut Microbiota and Its Metabolites on the Efficacy of Immunotherapy in Metastatic Colorectal Cancer  NCT06714903 | Metastatic colorectal cancer | Observational, prospective study  Group 1: sintilimab plus fruquintinib  Group 2: fruquintinib alone  Variables collected: GM; metabolomic and proteomics signatures | - IO efficacy | - |
| The Gut Microbiome and Immunotherapy Response in Solid Cancers  NCT06050733 | Solid cancer patient | Observational, Cross-sectional  Group 1: patients with disease progression  Group 2: patients with stable or experience shrinkage in tumour size | - Characterization of fecal microbiome | - Cognitive function - Fatigue - Gastrointestinal health |
| The Intestinal Microbiome in Triple Negative Breast Cancer Treated with Immunotherapy (IMPACT)  NCT06318507 | Breast Neoplasms | Observational, prospective study  Variables collected: GM diversity | - pCR | - |
| ARGONAUT: Stool and Blood Sample Bank for Cancer Patients  NCT04638751 | - NSCLC - TNBC - Colorectal cancer - Pancreatic cancer - High risk for colorectal cancer | Observational, prospective study | - GM predictiveness of PFS - GM predictiveness of colorectal cancer | - Correlation between GM composition and immune markers - GM predictiveness of OS - Library building |
| Gut Microbiome and Treatment for Gynaecological Cancer Patients Receiving Immunotherapy  NCT04957511 | Advanced or recurrent gynaecological cancer | Observational, prospective study | - GM microbiome changes | - |
| Association Between Microbiome and the Efficacy and Safety of PD-1/PD-L1 Blockade in Resectable NSCLC  NCT06613308 | Resectable NSCLC | Observational, prospective study  Group 1: Neoadjuvant IO+ CTx  Group 2: Neoadjuvant CTx | - mPR - pCR | - DFS - OS - irAEs - Microbes in respiratory and gut tracts - Radiological response - Single-cell immune repertoire |
| Development and Analysis of a Stool Bank for Cancer Patients  NCT04291755 | Patients undergoing cancer IO | Observational, prospective study  Variables collected: Stool, blood, urine samples | - ICIs response |  |
| Microbiome Immunotherapy Neoadjuvant Assessment (MINA)  NCT06709651 | early-stage TNBC | Observational, prospective study | - Local breast cancer microbiome pre-vs post-therapy | - Local breast cancer microbiome and pCR - Local breast cancer microbiome and event-free survival - Local breast cancer microbiome and OS - Local breast cancer microbiome and TILs - GM pre- vs post-therapy |
| Microbiome Immunotherapy Toxicity and Response Evaluation  NCT04107168 | Advanced cancer | Observational, prospective study | - GM predictiveness of PFS | - GM predictiveness of OS - GM predictiveness of relapse - GM correlation with treatment efficacy - GM correlation with incidence and type of irAEs - GM correlation with patients features - Library building |
| Modulation of the Gut Microbiome With Pembrolizumab Following Chemotherapy in Resectable Pancreatic Cancer  NCT05462496 | Pancreatic adenocarcinoma | Single-arm interventional study  Intervention: antibiotics+pembrolizumab following CTx | - Overall immune response | - AEs incidence - R0 resection rate - Proportion of participant with histologic regression score 0, 1 or 2 - ORR - OS |
| A Study of Oncobax®-AK in Patients With Advanced Solid Tumors  NCT05865730 | NSCLC and RCC | Single-arm interventional study (phase 1/2)  Intervention: Live bacteria product: Akkeremansia municiphila | - ORR | - PFS |
| The Impact of Probiotic on Survival and Treatment Response in Metastatic Non-small Cell Lung Cancer Patients  NCT06428422 | Metastatic NSCLC | Randomized interventional study  Intervention: Bifidobacterium animalis subsp. Alctis B1-04  Control: placebo | - Clinical Response - PFS - OS | - GM modulation - Immunological findings |
| Metastatic Melanoma Patients on Immunotherapy With Nutritive Intervention Based on Mediterranean Diet (MINI-MD)  NCT06236360 | Metastatic melanoma | Randomized interventional study  Intervention: MedDiet  Control: no diet | - Levels of ingested flavones, anthocyanin; w-3 FA; Vitamin D; fiber | - Radiological response rate - Association between GM changes and IO response - QoL - Biochemical biomarker of melanoma (S100 and LDH) - GM changes and immune response and AEs |
| The Effect of Diet and Exercise on ImmuNotherapy and the Microbiome (EDEN)  NCT04866810 | Melanoma | Randomized interventional study  Intervention: High-fibre, plant-based diet+ exercise  Control: Standard Diet and exercise  Variables collected: GM signatures | - Feasibility | - OS - QoL - ORR |
| Prebiotic Food-enriched Diet (PreFED) to Enhance the Microbiome and Response to First-line Immunotherapy in Unresectable Melanoma  NCT06466434 | Unresectable melanoma | Single-arm interventional study  Intervention: Prebiotic Food-enriched diet | - Stool Faecalibacterium abbundances |  |
| High-Intensity Exercise and High-Fiber Diet for Immunotherapy Outcomes in Melanoma Patients: The DUO Trial  NCT06298734 | Advanced melanoma | Randomized interventional study  Arm 1: Exercise program  Arm 2: Diet program  Arm 3: Exercise+ diet program  Arm 4: no intervention | - GM diversity | - Systemic immune function - Cardiopulmonary fitness - Short Physical Performance Battery - Body composition - Anthropometric measures |
| FMT+ Immunotherapy+ Chemotherapy As First-line Treatment for Driver-gene Negative Advanced NSCLC  NCT06403111 | NSCLC | Single-arm interventional trial  Intervention: CTx + IO + FMT | - PFS | - ORR - AEs incidence - DOR - GM diversity - QoL |
| FMT to Convert Response to Immunotherapy  NCT05251389 | Advanced cutaneous end stage melanoma | Randomized interventional study  Intervention: FMT from a ICI non-responding donor  Control: FMT from a ICI responding donor | - Efficacy (SD, PR, CR | - Safety - PFS - GM changes and stability - Immune cells population changes in the TME |
| Fecal Microbiota Transplantation With Immune Checkpoint Inhibitors in Lung Cancer  NCT05502913 | Metastatic lung cancer | Randomized interventional study  Intervention: SoC [IO± CTx] + FMT  Control: SoC | - PFS | - OS - ORR - Rate of Disease Control - Microbiome analysis - Antibody and lymphocytes subpopulation - Safety and feasibility |
| Fecal Microbiota Preventing Toxicity in Renal Cancer Patients Treated With Immunotherapy Using Fecal Microbiota Transplantation (PERFORM)  NCT04163289 | RCC | Single-arm interventional study  Intervention: FMT | - Immune-related colitis occurrence | - irAEs incidence - Treatment discontinuation because of irAEs - ORR - GM changes - Immune response - QoL - PFS - OS - Tumor immune profile |
| Fecal Microbiota Transfer in Liver Cancer to Overcome Resistance to Atezolizumab/Bevacizumab (FLORA) (FLORA)  NCT05690048 | HCC | Randomized interventional trial  Intervention: FMT  Control: placebo FMT | - CD8 T-cell tumoral infiltration - AEs | - PFS - OD - Hepatic function |
| FMT in IT-refractory HCC - FAB-HCC Pilot Study  NCT05750030 | HCC | Single-arm interventional trial  Intervention: FMT+ Atezolizumab + Bevacizumab | - Safety (AEs incidence) | - Efficacy (CR, PR, SD, PD - Efficacy (ORR, DCR) - Efficacy (PFS, OS) - QoL - GM composition - Gut immune activity - Circulating immune cells - Stool metabolomics and lipidomics |
| Fecal Microbial Transplantation in Combination With Immunotherapy in Melanoma Patients (MIMic)  NCT03772899 | Melanoma | Single-arm interventional study  Intervention: FMT | - Safety | - ORR - GM composition - Immune blood biomarkers - metabolomics |

GM: Gut microbiome; pCR: pathological Complete Response; NSCLC: Non-Small Cell Lung Cancer; TNBC: Triple Negative Breast Cancer; PFS: Progression Free Survival; OS: Overall Survival; CTx: Chemotherapy; mPR: major Pathological Response; DFS: Disease Free Survival; IO: immunotherapy; TILs: Tumour Infiltrating Leukocytes; ORR: Overall Response Rate; RCC. Renal Cell Carcinoma; MedDiet: Mediterranean diet; w-3 FA: omega-3 Fatty Acid; QoL: Quality of Life; FMT: Fecal Microbiome Transplantation; DOR: Duration Of Response; SD: Stable Disease; PR: Partial Response; CR: Complete Response; TME: Tumor microenvironment; SoC: Standard of Care; HCC: Hepatocellular Carcinoma; PD: Progressive Disease.

**Table 3: Ongoing clinical trial investigating the association between alternative diets and immunotherapy.**

| **Title** | **Patients** | **Study design** | **Primary outcome/s** | **Secondary outcomes** |
| --- | --- | --- | --- | --- |
| FASTing-like Approach and Maintenance IMMunotherapy in ES-SCLC Patients Not Progressing on Chemo immunotherapy Induction (FASTIMMUNE)  NCT05703997 | ES-SCLC | Single-arm interventional study  Intervention: 5-day calorie restriction | - PFS | - OS - Compliance - AEs - Plasma amino acids - Plasma fatty acids - Serum growth factors - Peripheral blood immune cell populations |
| Low Dose TamOxifen and LifestylE Changes for bReast cANcer prevenTion (TOLERANT)  NCT06033092 | Women at increased risk for BC | Randomized interventional study  Arm 1: Low dose tamoxifen + Intermittent Caloric Restriction  Arm 2: Lifestyle intervention  Arm 3: Lifestyle Intervention + Intermittent Caloric Restriction  Arm 4: Low dose tamoxifen | - Post intervention levels of circulating binding globulin |  |
| Ketogenic Dietary Intervention to Improve Response to Immunotherapy in Patients with Metastatic Melanoma and Metastatic Kidney Cancer  NCT06391099 | Metastatic RCC and melanoma | Randomized interventional study:  Intervention: Ketogenic diet  Control: usual care | - Incidence of AEs - Feasibility |  |
| A Pilot and Feasibility Study of a Dietary Intervention with Low-protein Meals in Cancer Patients Receiving Immunotherapies  NCT05356182 | Cancer treated with immunotherapy | Randomized interventional trial  Intervention: Low-protein diet  Control: Control diet | - Feasibility | - Immune response - Safety and tolerability - Efficacy |

ES-SCLC: Extensive-stage small cell lung cancer; BC: Breast Cancer; RCC: renal cell carcinoma; AEs: Adverse Event.
